# Prevalence & Impact of COVID-19 in Systemic Sclerosis Patients and Assessment of the Demographic & Clinical Features in Cases Associated with Worse Prognosis: Results of a Single Centre Registry

**DOI:** 10.31138/mjr.34.2.172

**Published:** 2023-06-30

**Authors:** Hadi Poormoghim, Fatemeh GaffariRad, Shahrzad Rahmani, Negin Mohtasham, Simin Almasi, Ali Sobhani, Maryam Salimi-Beni, Elham Andalib, Parsa Amiri Naeini, Arash Jalali

**Affiliations:** Iran University of Medical Sciences, Tehran, Iran

**Keywords:** systemic sclerosis, COVID-19 prevalence, COVID-19 impact

## Abstract

**Background:**

Our knowledge of the COVID-19 infection impact on systemic sclerosis (SSc) is scarce. This study aimed to assess the prevalence of COVID-19 infection and to determine the predictive factors of worse outcomes and death in SSc patients.

**Methods:**

In this cohort study all patients who attended our clinic between 20^th^ February 2020 and 20th May 2021 were followed, and those with a history of COVID-19 infection completed the questionnaire. Results of para-clinical tests were extracted from the SSc database. The outcomes were classified as: alive vs. deceased and, mild vs. worse outcomes. Descriptive statistics and binary logistic regression models were applied.

**Results:**

Of the total 192 SSc patients studied, COVID-19 affected 12.5%; 6% experienced mild disease, 7% were hospitalized and 3% died. The worse outcome was associated with: older age [95%CI: 1.00–1.08], smoking [95%CI: 2.632–33.094], diabetes [95%CI: 1.462–29.654], digital pitting scars (DPS) [95%CI: 1.589–21.409], diffusing capacity of the lungs for carbon monoxide [DLCO<70 [95%CI: 1.078–11.496], left ventricular ejection fraction (LVEF)<50% [95%CI: 1.080–38.651], systolic pulmonary artery pressure (sPAP)>40 mmHg [95%CI: 1.332–17.434], pericardial effusion (PE) [95%CI: 1.778–39.206], and tendon friction rub [95%CI: 1.091–9.387]. Death was associated with male gender [95%CI: 1.54–88.04], hypertension [95%CI: 1.093–2.155], digital ulcers (DU) [95%CI: 0.976–18.34], low forced vital capacity (FVC) [95%CI: 0.03–0.81], and joint flexion contracture (JFC) [95%CI: 1.226–84.402].

**Conclusion:**

Risk factors for the worse outcome in COVID-19 infected SSc patients included, older age, smoking, diabetes, DPS, DLCO<70, LVEF<50%, sPAP>40 mmHg, PE, and TFR. Death was associated with the male gender, hypertension, DU, low FVC, and JFC.

## INTRODUCTION

In December 2019, an outbreak of a viral acute respiratory illness emerged in Wuhan, China, causing a type of severe acute respiratory syndrome, which was named ‘COVID-19’. On March 11^th^ 2020, the World Health Organization (WHO) declared the novel corona-virus (COVID-19) outbreak a global pandemic.^1^

The COVID-19 outbreak has become a global priority for healthcare systems since 2020 and has also affected patients without COVID-19 infection. In patients with systemic sclerosis, COVID-19 has affected both the diagnosis and management of the disease. The COVID-19 outbreak also compromised certain clinical studies that had begun before the pandemic.^2^

In a systematic review that addressed the prognostic factors of COVID-19 patients, older age, the male sex, smoking and comorbidities such as diabetes, hypertension, CVA, COPD, chronic renal diseases, and cardiovascular diseases were associated with an increased risk of severe COVID-19 disease and related mortalities.^3^

Certain studies have been conducted on the impact of the COVID-19 outbreak on SSc patients by the World Scleroderma Foundation’s EUSTAR (European Scleroderma Trials and Research group), and in different countries since the beginning of the COVID-19 pandemic. The prevalence of COVID-19 among SSc patients was higher than in the general population in Italian studies, and was also higher in patients with symptomatic interstitial lung disease (ILD).^4^ In the EUSTAR study, more severe prognosis was observed in COVID-19 infected SSc patients who were older, had ILD, renal disease, and non-scleroderma comorbidities.^5^

Few studies have referred to the prevalence and mortality impact of COVID-19 in SSc patients during the waves of disease. In conclusion, we decided to conduct a study on the aforementioned topics.

The aim of this study was to describe the prevalence of COVID-19 on SSc patients and to determine the patients’ clinical features associated with worse outcomes and mortality in a cohort study conducted on geographical areas with a high frequency of infection.

## METHODS

This cohort study included all patients who had attended our scleroderma clinic at Firoozgar Hospital, Tehran, Iran, from 20^th^ February 2020 to 20th May 2021. All SSc patients in this study had been diagnosed according to the ACR/EUSTAR criteria^6^ and disease subset distinction was made based on Le Roy’s criteria.^7^

A history of infection with COVID-19 was taken from all patients who visited the clinic. We contacted those who had cancelled their appointments through phone calls. The patients were included in the study as COVID-19 cases if they had the typical COVID-19 symptoms with one of the following findings, a) positive PCR, b) positive antigen rapid test (Ag-RDT), or c) a positive PCR among family members, and d) those with typical symptoms and COVID-19–related HRCT findings. All patients with a positive history of COVID-19 completed the questionnaire used in the EUSTAR COVID-19 study (with some modifications). The patients were excluded from the study if they had fever, diarrhoea, dyspnoea related to systemic sclerosis complications, infected ulcers, small intestinal bacterial overgrowth (SIBO), or diffuse lung fibrosis.

Patients’ data were extracted from the database used in our cohort. The last available ILD diagnosis results following HRCT, PFT and DLCO (performed within the last 12 months) were used. The disease spectrum was defined as mild and severe/critical–as recommended by the WHO.^8^

We classified the outcomes into four categories in our study: alive vs. dead, and mild vs. severe/critical illness. Mild illness was defined as when patients had mild symptoms and did not require hospitalization and stayed at home. Severe/critical illness was defined as when patients needed hospitalization due to severe dyspnoea and required oxygen therapy by non-invasive ventilation or mechanical ventilation.

### Statistical methods

Categorical data as well as frequencies and percentages were compared using the Chi-square test or Fisher’s exact test. T-tests or Mann-Whitney U tests were used to analyse continuous variables. To determine the association between organ involvement and worse prognosis, binary logistic regression analysis was used to calculate the odds ratio (OR). Differences were considered significant when p<0.05. STATA software was used to draw the forest plot.

### Ethical issues

Ethical approval was obtained from the Human Research Ethics Committee in our institution [Ethics code: IR.IUMS. FMD.REC.1400.511]. The study was conducted in accordance with the principles of the Declaration of Helsinki.

## RESULTS

Of the 192 patients with systemic sclerosis, 24 (12.5%) patients were infected with the COVID-19 virus, 174 (91%) were female, 97 (51%) had dcSSc. The main clinical, demographic, and serological features and treatments of the two groups of SSc patients prior to the infection are shown in **Table 1**.

**Table 1.** Demographic and baseline data in SSc patients without COVID-19 and with COVID-19.

	**SSc patients not affected with COVID-19 = 168**	**SSc patients affected with COVID-19 = 24**

Age [Mean (SD)]	49.3 (11.9)	55.83 (12.7)
Gender (male)	15 (8.9)	3 (12.5)
Disease subsets: diffuse	87 (51.8)	11 (45.8)
Cigarette Smoking	5(2.9%)	9 (37.5)

**Comorbidities**		
BMI [Mean (SD)]	25.7 (4.5)	26.8 (4.54)
Diabetes No: Total	4:155 (2.6)	5:24 (20.8)
Hypertension BP>130/90	19 (11.6)	4 (16.7)

**Modified Rodnan Skin Score**	12.1±8.3	13.4 ±8.9

**Vascular lesion**		
Telangiectasia	121 (72.0)	18 (75.0)
Digital pitting	69 (41.1)	15 (62.5)
Digital ulcer	47 (28.0)	10 (41.7)
Digital gangrene	4 (2.4)	3 (12.5)
Calcinosis	42 (25.0)	6 (25.0)

**GI symptoms**		
Reflux	150 (89.3)	18 (75.0)
Bloating	11 (6.5)	2 (8.3)
Diarrhea	32 (19.0)	4 (16.7)

**Lung disease (ILD)**		
FVC<70%	54 (32.1)	11 (46)
DLCO<70%	73 (45.1)	13 (54.2)

**Cardiac involvement #**		
Ejection Fraction <50%	5:163 (3.1)	1:24(4.2)
PAP >40	12:162 (7.4)	4 :24(16.7)
Diastolic dysfunction	91:163 (55.8)	18:24 (75.0)
Pericardial effusion	6:163 (3.7)	2:24 (8.3)

**Musculoskeletal system**		
Arthritis >= 1	35 (20.8)	9 (25.0)
Flexion contracture	71 (42.3)	11 (45.8)
Muscle weakness	21 (12.5)	6 (25.0)
Myalgia	25 (14.9)	2 (8.3)
Tendon friction rub	39 (23.2)	6 (25.0)

**Kidney involvement (Hx of SRC)**	5 (4.2)	2 (8.7)

**Autoantibodies #**		
ANA	144:178 (92.3)	20 (90.9(
Anti-TOPO	108:178 (68.8 )	15 (71.4)
ANTI-Centromere	17:175 (11.0)	2 (9.5)

**Medications** administered prior to COVID-19 infection		
Non cytotoxic	50 (29.8)	5 (20.8)
Mycophenolate	80 (47.6)	14 (58.3)
Preednisolone	134 (79.8)	20 (83.3)

# denominator of the factors is the number of patients who have been tested.

The mean age of SSc patients affected with COVID-19 was higher than those who were not affected [55.83 ± 12.7 vs. 49.3 ± 11.9; p=0.012)]; 5.4% vs. 21% (p=0.006) were active smokers. Likewise, significant differences were seen in COVID-19 infected patients vs. non-infected patients in terms of comorbidities such as, diabetes [5 (20.8%) vs. 4 (2.6%); p=0.0001], digital gangrene [3 (12.5%) vs. 4 (2.4%); p=0.013], and cardiac, lung, gastrointestinal, and renal involvement. Autoantibody profile and treatment options showed no significant effects on susceptibility to COVID-19 infection.

### COVID-19 symptoms in SSc patients

The most prevalent COVID-19 symptoms in our study were malaise/fatigue (91.7%) and fever (88.0%). Myalgia was reported in 41.5% and respiratory symptoms such as dyspnoea (leading to oxygen inhalation) were reported in 42.0% and 29% of cases, respectively. Eight patients (33%) reported diarrhoea, seven (29.0%) reported smell & taste disturbance and 2% reported rhinorrhoea and sneezing (**Table 2**).

**Table 2.** COVID-19 symptoms in 24 patients with SSc.

**Symptoms**	**No (%)**
**General symptoms**	
Fever > 37.5	21 (88.0)
Fatigue/Malaysia	22 (92.0)
Headache	1 (4.0)
**Musculoskeletal symptoms**	
Myalgia	10 (42.0)
**Ear, Nose, Throat**	
Conjunctivitis	0
Cough/sputum	9 (38.0)
Rhinorrhoea/sneezing	2 (8.0)
Odour/taste disturbance	7 (29.0)
**Respiratory symptoms**	11 (46.0)
Dyspnoea	10 (42.0)
Need Oxygen during COVID-19 infection	9 (38.0)
**Gastrointestinal Symptoms**	
Diarrhoea	8 (33.0)

### COVID-19 outcomes in SSc patients

Of the total 192 SSc patients COVID-19 infection occurred in 24 (12.5%) patients, 11 (5.7%) had mild disease and stayed at home until recovery. The worse outcome (severe/critical illness that required oxygen/mechanical ventilation) occurred in 13 (6.7%) patients and death occurred in 6 (3.1%) patients. Five deaths were the result of COVID-related pneumonia, and one was due to CVA.

### COVID-19 outcome within the infected group

Details of data related to the exposure, diagnosis, care setting, outcome, and treatment in the COVID-19 infected group of SSc patients are shown in **Table 3**. The data are based on the outcomes. Within the infected group, 46% had mild disease, 54% had severe/critical status, 75% recovered and 25% passed away.

**Table 3.** SSc patients’ data on exposure, diagnosis, setting of care, outcome, and COVID-19 treatment based on outcome.

	**All SSc infected patients**	**Mild vs worse outcome**		**Alive vs death outcome**	

		Mild disease Treated at home	Need oxygen / mechanical ventilation (Severe/critical)	**Alive No(%)**	**Died No(%)**

**SSc patients infected with COVID-19**	24	11 (46)	13 (54.0)	18 (75)	6 (25.0)

**History of exposure**					
Contact with a household member	14 (58.3)	6 (54.0)	8 (62.0)	11 (61.1)	3 (50.0)
Presence in public events	1 (4.2)	0	1 (8.0)	0	1 (17.0)
Unknown	9 (37.5)	5 (45.0)	4 (31.0)	7 (39.0)	2 (33.3)

**Diagnosis based on symptoms and:**					
HRCT	1 (4.0)	0	1 (8.0)	-	1 (17)
PCR	17 (71.0)	8 (72.0)	9 (70.0)	13 (72.0)	4 (67.0)
PCR in sibling	1 (4.0)	1 (9.0)	0	1 (6.0)	0
Rapid test	5 (21.0)	2 (18.0)	3 (23.0)	4 (22.0)	1 (17.0)

**Setting of care**					
Home isolation	11 (46.0)	11 (100.0)	0	11 (61.1)	0
Hospitalisation	13 (54.0)	0	11 (85.0)	7 (39.0)	6 (100.0)
ICU	2 (8.0)	0	2 (15.0)	1 (6.0)	1 (17.0)

**Treatment of COVID-19 disease**					
Antiviral	12 (50.0)	3 (27.0)	9 (70.0)	7 (39.0)	5 (83.0)
Antirheumatic drug (HCQ/Colchicine)	4 (17.0)	3 (27.0)	1 (8.0)	3 (17.0)	1 (17.0)
Antibiotics	11 (46.0)	10 (91.0)	1 (8.0)	10 (56.0)	1 (17.0)
HCQ	4 (17.0)	1 (9.0)	0	3 (17.0)	1 (17.0)
Convalescent Plasma Therapy	1 (4.0)	0	1 (8.0)	1 (16.0)	0
Steroids	12 (50.0)	8 (72.0)	4 (31.0)	8 (44.0)	4 (67.0)
Ventilatory support (oxygen required)	10 (42.0)	5 (45.0)	5 (38.0)	5 (28.0)	5 (83.0)

Required oxygen before COVID-19	2 (8.0)	0	2 (15.0)	1 (16.0)	1 (17.0)
Required oxygen during COVID-19 treatment	9 (38.0)	2 (18.0)	7 (54.0)	5 (28.0)	4 (67.0)

**Mycophenolate before COVID-19 infection**	14 (58.0)	6 (65.0)	8 (62.0)	11 (61.0)	3 (50%)
**Mycophenolate** during covid-19 course **Ŧ**	8 (57.0)	5 (83.0)	3 (38.0)	7 (64.0)	1 (33.0)

Ŧ In the last row, the numerator is the number of patients on mycophenolate before the COVID infection.

### COVID-19 diagnosis

The COVID-19 diagnosis was based on both the symptoms and the PCR test result in 17 (70.8%) patients. In one patient the diagnosis was based on typical COVID-19 symptoms and a positive PCR among family members. Another patient was diagnosed based on typical symptoms and COVID-19 – related HRCT findings.

In 5 symptomatic patients, the diagnosis was based on the positive antigen rapid diagnostic test (Ag-RDT) result.

### Exposure status

Fourteen of the 24 COVID-19 patients (58%) had had contact with household members; 1 (4.2%) had been present in public events, and in 9 patients the source of exposure was unknown.

### Care setting

Eleven (45.8%) patients were isolated at home, and 13 (54.2%) were hospitalized, 2 (8.3%) of which were admitted to the ICU.

### Treatment

Twelve (50%) patients were treated with antiviral drugs (3 with Favipiravir, 8 with Remdesivir, and 1 with Sofosbuvir). The remaining drugs administered to the patients were as follows: antirheumatic drugs (Hydroxychloroquine/Colchicine) to 11 (45.8%), antibiotics to 4 (4.3%), Hydroxychloroquine to 1 (4.2%), convalescent plasma therapy to 1 (4.2%), steroids to 12 (50%), and oxygen/ventilation support to 10 (42%) patients.

### Treatment of SSc patients before and during the course of infection with COVID-19

Before being infected with COVID-19, two (8.3%) SSc patients needed oxygen for extensive ILD. During the course of the infection, oxygen therapy was needed in 10 (42%) patients.

Twenty (83.3%) patients were treated with low dose prednisolone (<10 mg); 14 (58%) patients had taken mycophenolate mofetil (MMF) before the infection. Eight (62%) patients in the group with the worse outcome (n=13), and six (55.0%) in the group with mild outcome (n=11) were on MMF, the difference of which was not significant. MMF continued to be administered throughout the course of infection in 8 of 14 (33%) patients. The worse outcome ensued in 3 of 13 (23%) and the mild outcome ensued in 5 of 11 (45%) patients. Three of 6 (50%) and 11 of 18 (61%) patients died.

### Factors associated with severe outcome (death, ICU, ventilation)

**Figure 1.**
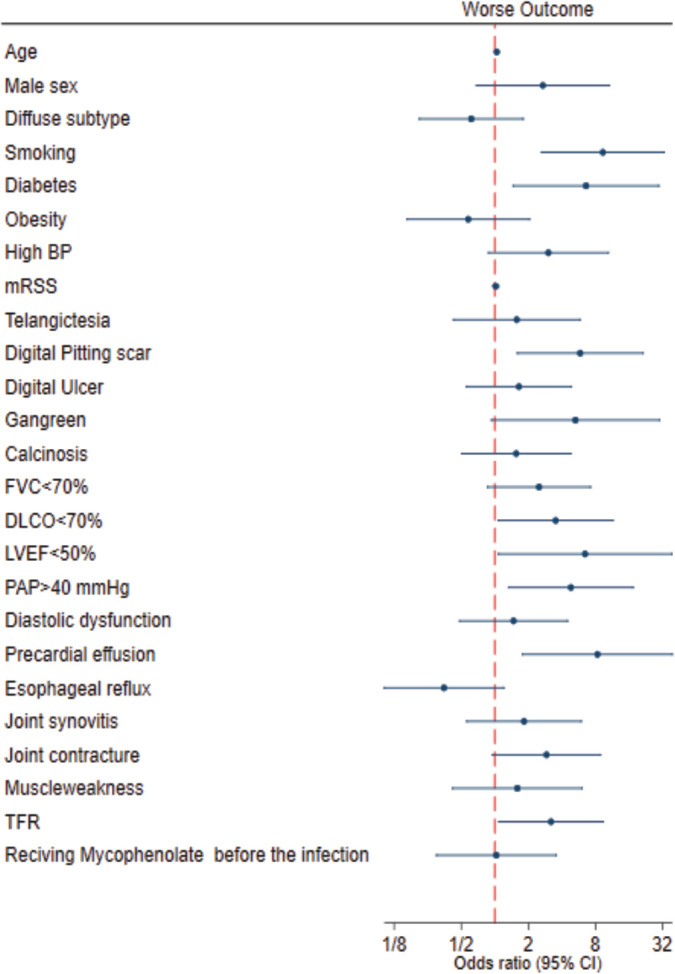
Forest plot shows univariate analysis of demographic, comorbidity, clinical, and serology features with worse outcome in 24 SSc-patients infected with COVID-19.

The worse outcome in SSc patients with COVID-19 showed associations with older age [OR: 1.042 (95% CI: 1.00–1.08), p=0.05] and non-scleroderma comorbidities such as smoking [OR: 9.333 (95% CI: 2.632–33.094), p=0.001] and diabetes [OR: 6.583 (95% CI: 1.462–29.654), p=0.01]. Scleroderma related factors that affected the worse outcome included, digital pitting scar [OR: 5.833 (95% CI: 1.589–21.409), p=0.008], DLCO<70 [OR: 3.520 (95% CI: 1.078–11.496), p=.037], LVEF<50% [OR: 6.462 (95% CI: 1.080–38.651), p=0.04], systolic pulmonary artery pressure (sPAP)>40 mmHg, [OR: 4.818 (95% CI: 1.332–17.434), p=0.017], pericardial effusion [OR: 8.350 (95% CI: 1.778–39.206), p=0.007], and tendon friction rub (TFR) [OR: 3.201 (95% CI: 1.091–9.387), p=.034]. Other clinical features or demographic data, autoantibodies, and medications were not associated with the worse outcome over the course of the disease (**Figure 1**).

**Figure 2.**
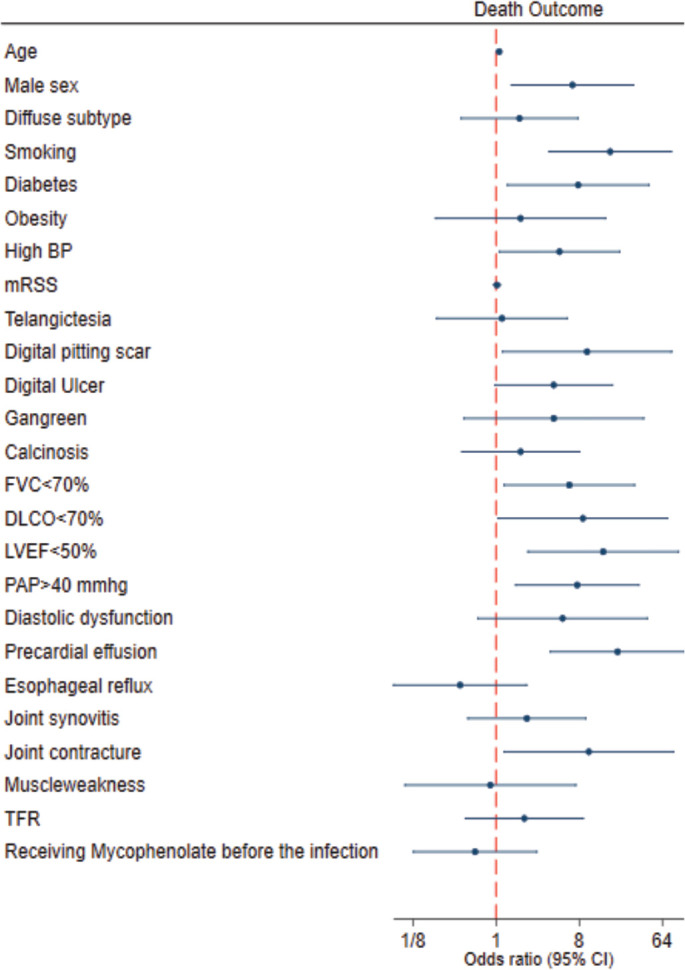
Forest plot shows univariate analysis of demographic, comorbidity, clinical, and serology features with death outcome in 24 SSc-patients infected with COVID-19.

Death in SSc patients with COVID-19 was associated with age [OR: 0.93 (95% CI: 0.87–0.98), p=0.007], the male gender [OR: 37.0 (95% CI: 1.54–88.04), p=0.02], and non-scleroderma comorbidities; smoking [OR: 17.40 (95% CI: 3.785–79.99), p=0.0001], hypertension [OR: 17.0 (95% CI: 1.29–223.14), p=0.03], and diabetes [OR: 7.810 (95% CI: 1.330–45.852), p=0.23]. The following scleroderma related factors were associated with increased risk of death: digital pitting scar [OR: 9.727 (95% CI: 1.17–80.68), p=0.03], digital ulcer [OR: 4.231 (95% CI: 0.976–18.34), p=0.05], low FVC<70% [OR: 1.60 (95% CI: 0.03–0.81), p=0.03 ], DLCO<70% [OR: 8.77 (95% CI: 1.05–72.8), p=0.04], LVEF<50%, [OR: 14.53 (95% CI: 2.220–95.79), p=0.05], pericardial effusion [OR: 20.88 (95% CI: 3.87–112.59), p=0.001], and joint contractures [OR: 10.17 (95% CI: 1.226–84.402), p=0.03] (**Figure 2**).

Disease subsets and mRSS, history of scleroderma renal crisis, anti-TOPO I positive antibodies or MMF intake before COVID-19 infection were not associated with worse or death outcomes (Supplementary table).

## DISCUSSION

Of 192 SSc patients, 24 (12.5%) were affected with COVID-19. Based on the main findings of the current study, COVID-19 infection occurred more commonly among older age groups, the male gender, cigarette smokers, and diabetics. The most frequent symptoms of COVID were fever/malaise, myalgia, respiratory symptoms, and diarrhoea. In our study, 24 (12.5%) patients were infected with COVID-19, and 6 (25%) of them passed away. Of the 24 COVID-19 infected patients 11 (46%) and 13 (54%) experienced mild and worse outcomes, respectively.

The worse outcome in COVID-19 affected SSc patients was associated with older age, smoking and diabetes. Scleroderma related factors that affected the worse outcome included, digital pitting scar, DLCO<70, LVEF<50%, sPAP>40 mmHg, pericardial effusion, and TFR. Death was associated with older age, the male gender, high blood pressure, active cigarette smoking, and characteristic SSc symptoms, such as, digital pitting scar, FVC and DLCO <70%, and digital flexion contracture.

### The prevalence of disease

In our study, the prevalence of COVID-19 was 12.5% and the COVID-19 specific mortality rate among SSc patients was 3.1%; the mortality was higher than COVID related mortality reported among the general population (prevalence: 6.8%; mortality: 0.07%).^9^ The case fatality rate among the COVID-19 infected SSc patients was 25%, while it was 3.7% among the general population. Factors such as, the background disease and immunosuppressive medications have been speculated as risk factors of infection in systemic sclerosis patients.^3,10^

In a large Italian study conducted on patients with rheumatic autoimmune systemic diseases affected by COVID-19, a higher prevalence of infection was reported in patients with connective tissue diseases (CTD) when compared to patients with inflammatory arthritis. Immune system dysfunction in patients with CTD has been postulated as a cause of this difference.^11^

Studies conducted on SSc patients in the first wave of the COVID-19 pandemic showed promising results, wherein no increase in their susceptibility to disease was observed.^12,13^ This finding may be explained as such, that patients were aware of their immunocompromised status and this fear led them to adhere to the health system’s recommendations, such as mask-wearing and protection during the early COVID-19 outbreak.^13,14^

Secondly, it has been speculated that in SSc patients MMF causes the release of pro-inflammatory cytokines by inhibiting T17, and thus, may play a protective role against COVID-19 infection, resulting in a low prevalence of the disease.^12,13^ However, we observed an increased risk of COVID-19 infection and mortality in SSc patients. The reason behind this dissimilarity could be the fear of the newly emerging virus at the beginning of the pandemic and subsequent strict adherence to mask-wearing. Fewer virulent subtypes of the virus may have also played a role here.^13,14,15^ Furthermore, we visited the patients for longer periods of time, starting from the first wave of the epidemic to the middle of the fourth wave in our country, so the results may depict a more realistic estimate of the COVID-19 impact on systemic sclerosis.

### Risk factors of worse disease and death

A comprehensive study by Izcovich et al indicated that older age, the male gender, smoking, hypertension, diabetes and underlying pulmonary, cardiovascular, renal, and cerebrovascular diseases were associated with increased risk of severe COVID-19 and mortality.^3^ Although this systematic review has not addressed scleroderma patients, it underlines the general risk factors for severity and mortality in COVID-19 disease. We too identified risk factors such as, age, the male gender, and smoking. Few studies have addressed the impact of COVID-19 on SSc patients. One Italian cohort study indicated that COVID-19 does not affect the occurrence, severity, morbidity, and mortality of SSc among these patients.^13^ Elsewhere, Hoffman-vold et al. learnt that worse outcomes were associated with older age, non-SSc comorbidities, SSc related renal disease or ILD*. (5)* Similarly, in our cohort, older age, and non-scleroderma comorbidities (diabetes, active smoking), and scleroderma related symptoms (digital pitting scar, DLCO<70, LVEF<50%, sPAP>40 mmHg, pericardial effusion, TFR) were associated with a more severe outcome. However, SSc related renal symptoms were not risk factors for a severe outcome.

There are some differences between the EUSTAR study and the current study’s findings. The EUSTAR study (Hoffmann) takes into account two outcomes: hospitalisation and severe outcome (defined as either non-invasive ventilation, mechanical ventilation/extracorporeal membrane oxygenation or death).^13^ In addition to the outcomes examined in this study, we investigated the death outcome as well. In our study, an increased risk of death was associated with age, the male gender, hypertension, disease related factors such as digital pitting scar and digital ulcers, low FVC, DLCO, and flexion contracture.

### Cytotoxic medicine

Although the prevalence of COVID-19 infection was higher in SSc patients when compared to the normal population, the prior use of MMF did not affect susceptibility to COVID-19 and did not prove to be a risk factor for worse outcome or death.

It was thought that immunosuppressive medications would increase the risk of infection in patients using these medications, and that morbidity and mortality would be higher in COVID infected patients on immunosuppressive medications. However, growing data indicate that this may not be the case. Similar to other studies and the EUSTAR study’s results, we too observed that using cytotoxic medication is not a risk factor for worse outcome in COVID-19–infected patients.^16,17^

### Strengths and limitations

The power of our study lies in its adherence to the ICD-10, which has been done to make the article useful for systematic reviews or comparative studies. Following the COVID-19 outbreak, various studies published throughout the world tried to reach a common definition of the disease in semantic terms. The result has been a new definition in the emergency ICD-10 code for COVID-19, to provide researchers with a common case definition of COVID-19, the latter of which has been released by the WHO as well.^18^

This study was a prospective cohort and the limited numbers of COVID-19 infected patients was an invariable factor. Moreover, due to the difference in virulence of the virus strains and treatment protocols in the four pandemic waves of the disease, the results were reported as cumulative prevalence rates. Multivariate analysis was impossible to conduct to determine the predictive factors of death given the few numbers of deceased patients.

## CONCLUSION

Overall, the prevalence of COVID-19 was higher in SSc patients than in the normal population. Moreover, it was associated with higher morbidity and mortality. Older age, the male gender, and cigarette smoking were predictive of death in SSc patients with COVID-19. Immunosuppressive medications had no effect on the severity of the disease and its mortality.
